# Survival of *Burkholderia pseudomallei* in distilled water for 16 years

**DOI:** 10.1016/j.trstmh.2011.06.004

**Published:** 2011-10

**Authors:** Apinya Pumpuang, Narisara Chantratita, Chanthiwa Wikraiphat, Natnaree Saiprom, Nicholas P.J. Day, Sharon J. Peacock, Vanaporn Wuthiekanun

**Affiliations:** a Department of Microbiology and Immunology, Faculty of Tropical Medicine, Mahidol University, 420/6 Rajvithi Road, Bangkok 10400, Thailand; b Mahidol-Oxford Tropical Medicine Research Unit, Faculty of Tropical Medicine, Mahidol University, Bangkok, Thailand

**Keywords:** *Burkholderia pseudomallei*, Melioidosis, Survival in water, Genetic alteration, Colony morphology, PFGE

## Abstract

*Burkholderia pseudomallei* was examined after being maintained in distilled water at 25 °C for 16 years. The Gram stain was atypical (pale pink cocci or coccobacilli). The estimated number of live and dead *B. pseudomallei* was 3.8 × 10^7^ cells/ml and 1.4 × 10^5^ cells/ml, respectively. A colony count on agar of 1.0 × 10^6^ cfu/ml suggested that a proportion of cells were in a viable but non-culturable state. Colony morphology was different from the parental isolate for 84% of colonies. Pulsed-field gel electrophoresis analysis of *Avr*II DNA restriction fragments revealed six different but related banding patterns, which may represent genomic rearrangement.

## 1 Introduction

The Gram-negative, non spore forming bacillus *Burkholderia pseudomallei* is the cause of melioidosis and classified by CDC as a Category B select agent. *Burkholderia pseudomallei* is present in the environment in northern Australia and across much of southeast Asia, where human infection is acquired by bacterial inoculation, inhalation or ingestion.^1^, ^2^ In the absence of a vaccine, strategies for the prevention of melioidosis are based on reduction of exposure. These could potentially include efforts to reduce the bioburden of *B. pseudomallei* in specific environments, including clean-up operations in geographic areas that have become contaminated for the first time through accident or bioterrorist activity. This is likely to be hampered, however, by the extreme hardiness of this organism. In 1995, we reported that *B. pseudomallei* strain E32 had survived in distilled water (DW) for three years.^3^ Here, we extend these observations and report on the survival and preliminary characterisation of a strain of *B. pseudomallei* maintained in DW at 25 °C for 16 years.

## 2 Materials and methods

*Burkholderia pseudomallei* strain 207a was isolated in 1986 from blood taken from a rice farmer presenting to Sappasithiprasong Hospital in northeast Thailand, and stored in trypticase soya broth (TSB) with 15% glycerol at –80 °C. In 1994, the organism was sub-cultured onto Columbia agar and inoculated into 9 ml DW to obtain 3.0 x 10^10^ cfu/ml contained in a plain plastic tube with a screw cap that was tightened and then loosened by a half turn. This was maintained in a cupboard at 25 °C. In December 2008, the volume was noted to be around 2.5 ml and DW was added once to a total volume of 15 ml. In January 2010, an aliquot of 5 ml was removed for the work described below.

## 3 Results and Discussion

Gram stain and light microscopy of bacilli from the original freezer vial demonstrated typical Gram-negative rods, while bacilli from DW were pale pink cocci or coccobacilli. The proportion of live versus dead bacteria in DW was defined using the LIVE/DEAD^®^ BacLight^TM^ viability stain according to the manufacturer's recommendations (Invitrogen, Carlsbad, California, USA). The estimated number of live and dead *B. pseudomallei* was 3.8 x 10^7^ cells/ml and 1.4 x 10^5^ cells/ml, respectively. Live bacteria were non-motile. A colony count was performed of the bacilli from DW on Ashdown agar (ASH) after serial dilution, spread plating, and incubation in air at 37 °C for four days. The count of 1.0 x 10^6^ cfu/ml was less than the estimated number of live bacteria using the viability kit, suggesting that a proportion of cells may be in a viable but non-culturable state.

**Figure 1:**
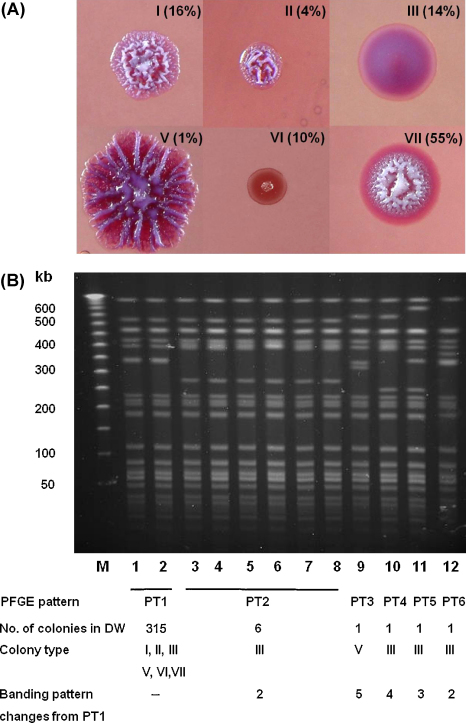
Colony morphology and pulsed-field gel electrophoresis (PFGE) banding pattern of *Burkholderia pseudomallei* strain 207a after being maintained in distilled water at 25 °C for 16 years. A total of 325 primary plate colonies were evaluated. (A) Six colony morphologies were identified on Ashdown agar; the percentage of each type is shown. All 236 primary plate colonies from the original freezer vial were Type I (data not shown). (B) Six related PFGE banding patterns (PT) were defined for the 325 DW colonies after *Avr*II restriction digest. The number of each PT, colony morphology and number of bands different from PT1 are shown. All 236 primary plate colonies from the freezer vial were PT1. M: ladder marker; Lane 1: Freezer vial control colony (FOC111-I); Lanes 2–12: water isolates (strain numbers DC219-I, DC6-III, DC73-III, DC122-III, DC234-III, DC257-III, DC323-III, DC69-V, DC86-III, DC258-III, DC253-III).

The entire original freezer vial (a volume of 80 μl) was subcultured onto ASH and incubated in air at 37 °C for four days. This resulted in a total of just 236 colonies, suggestive of cell death during freezing. Aiming to identify at least the same number of colonies for the DW sample, we performed serial dilution and spread plating of DW onto ASH and picked 325 individual, unselected colonies for further analysis. The colony morphology of the 236 colonies from frozen stock was uniformly type I, the characteristic ‘cornflower head’ appearance.^4^ The colony morphology of 325 colonies from DW were classified (in descending order of frequency) as: types VII, 55%; I, 16%; III, 14%; VI, 10%; II, 4%; and V, 1% (Figure 1).

Each of the 561 colonies was treated as an individual ‘strain’ and examined for lipopolysaccharide (LPS) pattern and banding pattern by pulsed-field gel electrophoresis. LPS was extracted and examined using SDS-PAGE and silver-staining, as described previously.^5^ The LPS pattern was a typical smooth type A for all 561 colonies. Pulsed-field gel electrophoresis (PFGE) using *Spe*I and *Avr*II was performed as described previously,^4^ and the banding patterns analysed using the BioNumerics software version 2.5 (Applied Maths, Sint-Martens-Latem, Belgium). The PFGE banding pattern of 236 freezer vial colonies showed no variability using either *SpeI* or *Avr*Il. The PFGE banding pattern of 325 DW colonies was identical using *SpeI*, but the *Avr*II restriction pattern revealed six different banding patterns. The *Avr*II restriction pattern for the freezer vial colonies was termed PT 1. A total of 315 DW colonies were also PT 1, while ten DW colonies had banding patterns that differed from the PT 1 pattern by 2 to 5 bands (Figure 1).

The morphological appearance of the 10 strains with altered PFGE banding patterns was type III (nine colonies) or type V (one colony). Reversible colony morphology switching of *B. pseudomallei* has been described in response to adverse environmental conditions.^4^ The 10 variant colonies each underwent seven serial subcultures in TSB and were then plated onto ASH. No change in colony morphology was observed, suggesting a fixed genetic event associated with alteration in the presence or function of one or more genes encoding a major surface expressed determinant.^4^

Our findings provide further evidence for the ability of *B. pseudomallei* to survive under extreme conditions. A proportion of colonies appeared to have undergone a putative genetic event based on PFGE banding pattern changes. This is the subject of further investigation.

## Author contributions

AP, NC, CW and NS performed the experimental work, data analysis and assisted in drafting the article. NC and SP designed the study protocol, interpreted the data and wrote the manuscript. ND and VW provided *B. pseudomallei* isolates, contributed to the conception of the study and critically reviewed the manuscript. All authors have read and approved the final manuscript.

## Funding

This study was funded by the Wellcome Trust, UK (grant number 089275/B/09/Z). NC holds a Wellcome Trust Career Development award in Public Health and Tropical Medicine.

## Conflicts of interest

None declared.

## Ethical approval

Not required.

## References

[1] Limmathurotsakul D., Wongratanacheewin S., Teerawattanasook N., Wongsuvan G., Chaisuksant S., Chetchotisakd P. (2010). Increasing incidence of human melioidosis in Northeast Thailand. Am J Trop Med Hyg.

[2] Wiersinga W.J., van der Poll T., White N.J., Day N.P., Peacock S.J. (2006). Melioidosis: insights into the pathogenicity of *Burkholderia pseudomallei*. Nat Rev Microbiol.

[3] Wuthiekanun V., Smith M.D., White N.J. (1995). Short report: Survival of *Burkholderia pseudomallei* in the absence of nutrients. Trans R Soc Trop Med Hyg.

[4] Chantratita N., Wuthiekanun V., Boonbumrung K., Tiyawisutsri R., Vesaratchavest M., Limmathurotsakul D. (2007). Biological relevance of colony morphology and phenotypic switching by *Burkholderia pseudomallei*. J Bacteriol.

[5] Anuntagool N., Wuthiekanun V., White N.J., Currie B.J., Sermswan R.W., Wongratanacheewin S. (2006). SC. Lipopolysaccharide heterogeneity among Burkholderia pseudomallei from different geographic and clinical origins. Am J Trop Med Hyg.

